# Gender disparities in time-to-initiation of cardioprotective glucose-lowering drugs in patients with type 2 diabetes and cardiovascular disease: a Danish nationwide cohort study

**DOI:** 10.1186/s12933-022-01713-3

**Published:** 2022-12-10

**Authors:** Kristian Løkke Funck, Lasse Bjerg, Anders Aasted Isaksen, Annelli Sandbæk, Erik Lerkevang Grove

**Affiliations:** 1 Steno Diabetes Center Aarhus, Aarhus University Hospital, Aarhus, Denmark; 2 Department of Public Health, Aarhus University, Aarhus, Denmark; 3 Department of Clinical Medicine, Health, Aarhus University, Aarhus, Denmark; 4 Department of Cardiology, Aarhus University Hospital, Palle Juul-Jensens Boulevard 99, 8200 Aarhus, Denmark

**Keywords:** Type 2 diabetes, Cardiovascular disease, Antidiabetic agents, Pharmacoepidemiology, Gender equity, Sex

## Abstract

**Background:**

We aimed to examine the impact of gender and specific type of cardiovascular disease (CVD) diagnosis (ischemic heart disease [IHD], heart failure, peripheral artery disease [PAD] or stroke) on time-to-initiation of either a sodium glucose cotransporter 2 inhibitor or glucagon-like peptide 1 analogue (collectively termed cardioprotective GLD) after a dual diagnosis of type 2 diabetes (T2DM) and CVD.

**Methods:**

In a nationwide cohort study, we identified patients with a new dual diagnosis of T2DM and CVD (January 1, 2012 and December 31, 2018). Cumulative user proportion (CUP) were assessed. Poisson models were used to estimate the initiation rate of cardioprotective GLDs. The final analyses were adjusted for potential confounders.

**Results:**

In total, we included 70,538 patients with new-onset T2DM and CVD (38% female, mean age 70 ± 12 years at inclusion). During 183,256 person-years, 6,276 patients redeemed a prescription of a cardioprotective GLD. One-year CUPs of cardioprotective GLDs were lower in women than men. Initiation rates of GLDs were lower in women (female-to-male initiation-rate-ratio crude: 0.76, 95% CI 0.72–0.81); adjusted 0.92, 95% CI 0.87–0.97). In CVD-stratified analysis, the adjusted initiation rate ratio was lower in female patients with IHD and heart failure (IHD: 0.91 [95% CI 0.85–0.98], heart failure: 0.85 [95% CI 0.73–1.00], PAD: 0.92 [95% CI 0.78–1.09], and stroke: 1.06 [95% CI 0.93–1.20]).

**Conclusions:**

Among patients with a new dual diagnosis of T2DM and CVD, female gender is associated with lower initiation rates of cardioprotective GLDs, especially if the patient has IHD or heart failure.

**Supplementary Information:**

The online version contains supplementary material available at 10.1186/s12933-022-01713-3.

## Introduction

Cardiovascular disease (CVD) is a major cause of premature morbidity and mortality in type 2 diabetes (T2DM) [1], and female T2DM patients face a higher risk of CVD compared with male patients [2]. Several biological and environmental factors may explain the uneven female-to-male risk ratio, and gender disparities in CVD treatment may be a factor that can be modified to even this disproportionate high CVD risk in women. Non-adherence to CVD medication is more common in women than men [3, 4], but whether lower drug initiation rates among females also contribute to increased CVD risk is unknown.

Two types of non-insulin glucose-lowering drugs (GLD: sodium glucose co-transporter 2 [SGLT-2] inhibitors and glucagon-like peptide 1 [GLP-1] analogues; collectively, cardioprotective GLDs), substantially reduce the risk of cardiovascular events and hospitalization for heart failure in T2DM patients with established CVD (ischemic heart disease, heart failure, peripheral heart disease or stroke) in clinical trials [5–11] and in real-world settings [12, 13]. Notably, treatment effects were of similar direction and magnitude in both genders [5–17] and, correspondingly, since 2017, the American and European guidelines have recommended either type of cardioprotective GLD irrespective of gender and type of CVD [18–20]. In 2019, EASD/ESC further emphasized that cardioprotective GLDs should be considered in patients with both CVD and T2DM irrespective of HbA1c level [20].

In Denmark, the time-to-initiation of cardioprotective GLDs in patients with a new dual diagnosis of T2DM and CVD has decreased since 2012 in parallel with the publication of major outcome trials and updated national guidelines [21], yet the proportion of patients with T2DM and CVD who initiate cardioprotective GLDs within one year after the dual diagnosis remains relatively low [21].

In this nationwide Danish cohort study, we examined time-to-initiation of cardioprotective GLDs in female and male patients with a dual diagnosis of T2DM and CVD between 2012 and 2018. Furthermore, we investigated if initiation rates differed according to type of CVD (ischemic heart disease, heart failure, peripheral artery disease [PAD] or stroke).

## Research design and methods

### Design

Population-based nationwide cohort study (Additional file 1: Fig. S1).

### Cohort

The study population was defined as all Danish adult patients with a first dual diagnosis of T2DM and CVD between January 1, 2012 and December 31, 2018, regardless of the diagnosis order (T2DM with new-onset CVD, or CVD with new-onset T2DM). Using a pre-specified algorithm, we identified all individuals with T2DM defined as individuals with ever use of any GLD (metformin, sulfonylureas, thiazolidinediones, dipeptidylpeptidase-4 inhibitors, GLP-1 receptor agonists except *Saxenda*, SGLT2 inhibitors or combination products; ATC codes for all drugs including GLDs are shown in Additional file 3: Table S1) or insulin use, any hospital diagnoses of diabetes or registration of screening/treatment for diabetes complications at a podiatrist, or laboratory results for HbA1c above 48 mmol/mol (6.5%) [22]. The date of the second registration of any of these events was considered the T2DM diagnosis date in order to account for any errors in the data and comply with diagnostic practice recommended by the World Health Organization (WHO) [23]. Patients with a majority of type 1 vs. type 2-specific diagnoses (ICD-10 codes DE10 vs. DE11) and insulin vs. non-insulin GLD purchases (ATC codes A10A vs. A10B) were considered to have type 1 diabetes and were excluded [22]. Also, women with gestational diabetes or polycystic ovaria syndrome were excluded.

CVD patients were defined as individuals with first occurrence of one or more International Classification of Diseases codes (ICD10) for ischemic heart disease, stroke, peripheral artery disease, or heart failure or associated procedural codes [24, 25] (Additional file 3: Table S2). The CVD diagnosis date was defined as the admission date from an inpatient admission with a primary or secondary code of CVD, or the first contact date in a hospital clinic outpatient course with a primary or secondary code of CVD.

The date at which patients received their second (latest) diagnosis (T2DM or CVD) according to the above definitions was defined as the index date of a dual diagnosis of T2DM and CVD in this study.

### Data sources

This cohort study is based on Danish registry data that has national coverage. The Danish health care system is publicly funded and the vast majority of health care services are free of charge. All health-related services are extensively documented at an individual level in national health care registries. In addition, Danish national registries hold information on routinely collected administrative data and contacts with social services. We included health care data from nationwide registries on drug prescriptions and diagnosis codes. Specifically, the Civil Registration System holds records of unique personal registry numbers for all Danish citizens since 1968. This registry was used to link prescription data from the National Database of Reimbursed Prescriptions [26] (complete data on prescriptions dispensed at community pharmacies in Denmark since 2004 up until 31 Dec 2018) and data on ICD diagnosis codes from the Danish National Patient Register (any hospital or outpatient contacts since 1977 and 1995, respectively, and with records up until 31 Dec 2018) [27]. Furthermore, we were able to extract detailed laboratory data from primary and secondary care in Denmark from the Register of Laboratory Results for Research and the Clinical Laboratory Information System of Central Denmark Region. In combination, the biomarker data covered all residents in Denmark from 2015 through 2018, and four of five Danish administrative regions were covered from 2011 onward.

### Variables

From the nationwide registries we retrieved information on: age, gender, demographic and educational level status, medications (cardiovascular medications including antihypertensives, antiplatelet and anticoagulant therapy, lipid-lowering drugs), diabetes duration (years since first recorded diabetes therapy), HbA1c, estimated glomerular filtration rate (eGFR), low-density lipoprotein (LDL) cholesterol, and cardiovascular comorbidity. The variables were obtained at the time of index date.

The study outcome was defined as the first dispense of any prescribed cardioprotective GLD (SGLT2-inhibitor or GLP1 analogue) after the onset of a dual diagnosis of T2DM and CVD. If a patient already received a cardioprotective GLD at inclusion, they were marked as current users.

### Statistical analysis

We tabulated characteristics of included individuals on the index date both overall and by sex [as mean (SD) for continuous data and as n (%) for categorical data]. Individuals with a double diagnosis before 2012 were excluded from the analysis. Patients who had not redeemed a cardioprotective GLD at any time (up to one year prior to the index date) were considered naïve for cardioprotective GLDs, while patients with prevalent use of GLDs were considered current users. Biochemical variables were identified as the closest measured value within 6 months before and 30 days after the index date.

We plotted the cumulative initiation proportion (CIP) curves and 95% CI for cardioprotective GLD both for CVD overall and stratified by cardiovascular complication type at inclusion (i.e., ischemic heart disease, heart failure, peripheral arterial disease, and stroke). As the study population is a high-risk population, we included competing risk of death in the CIP curves. The CIPs were plotted by sex. Furthermore, one-year cumulative user proportions (CUP) were estimated. Patients who were new or prevalent users of cardioprotective GLDs already on the index date of their dual diagnosis were included in the CIPs and CUPs, as we wanted to assess the overall likelihood of patients with T2DM and CVD receiving a cardioprotective GLD.

For cardioprotective GLD naïve patients, we used Poisson regression models to estimate crude cardioprotective GLD initiation rate (IRs), crude and adjusted initiation rate ratios (IRRs) with associated 95% confidence intervals (CIs), comparing female with male patients. The models were adjusted for age, diabetes duration, calendar year at inclusion, and education level (Model 1) as well as for HbA1c, LDL cholesterol, eGFR, antihypertensive drug use, and lipid-lowering drug use (model 2). We investigated if there was effect modification by sex on the association between CVD type and initiation rate using a Chi-Square test and stratified the analyses by type of cardiovascular disease. We further repeated the crude and fully adjusted analyses in four age strata.

Owing to the nature of the Danish registries, the dataset was largely complete, however especially for the biochemical variables, some missing variables were observed. Accordingly, we imputed missing data on covariates in 10 datasets using the Multiple Imputation by Chained Equations. Data were missing for up to 60% of individuals for variables (eGFR) used in the Poisson regression. Using Rubin’s rules, we summarized the obtained estimates from the Poisson models in the 10 imputed datasets. We performed a sensitivity analysis to assess any potential bias arising from imputation i.e., we assessed the association in a complete case analysis.

Statistical analyses were performed in R, version 4.1.3 (R Foundation for Statistical Computing, Vienna, Austria, www.R-project.org) using the Epi package and the Prodlim package for handling the data and analyses. The Multivariate Imputation by Chained Equations algorithm was used for imputation of missing data on covariates.

## Results

### Subjects

Among all Danish citizens, we identified 70,538 patients with a new dual diagnosis of T2DM and CVD between 2012 and 2018 (Table 1). Female patients compared to male patients were older, had lower education levels and, as part of their dual index diagnosis, they were more often diagnosed with T2DM first and CVD second. The distribution of CVD type differed between genders with a lower representation of ischemic heart disease in women and conversely, slightly larger proportions of heart failure, PAD and stroke in women. Small differences in HbA1c, LDL-cholesterol and eGFR were observed and prevalent drug use of a cardioprotective GLD at baseline was similar between men and women.

**Table 1:** Characteristics of female and male patients with a dual diagnosis of type 2 diabetes and cardiovascular disease 2012–2018

	All (n = 70.538)	Female (n = 27.133)	Male (n = 43.405)	Missing values
Age in years (mean, SD)	69.9 (11.9)	72.1 (12.3)	68.6 (11.3)	0.0
Diabetes duration in years (mean, SD)	3.7 (5.8)	4.1 (6.1)	3.5 (5.6)	0.0
Education level (n, %)				3.7^a^
0–10 years	26,358 (38.8)	12,556 (48.5)	13,802 (32.8)	
10–15 years	33,775 (49.7)	11,048 (42.6)	22,727 (54.1)	
> 15 years	7795 (11.5)	2305 (8.9)	5490 (13.1)	
HbA1c mmol/mol (mean, SD)	55 (16)	54 (15)	56 (16)	22.4^a^
LDL mol/l (mean, SD)	2.3 (1.0)	2.4 (1.1)	2.3 (1.0)	33.1^a^
Estimated GFR ml-min per 1.73m^2^ (mean, SD)	69 (22)	67 (22)	71 (21)	61.0^a^
Use of cardioprotective glucose-lowering drug prior to or initiated at index date (n, %)	3256 (4.6)	1184 (4.4)	2072 (4.8)	0.0
Use of SGLT2 inhibitor prior to or initiated at index date (n, %)	1005 (1.4)	329 (1.2)	676 (1.6)	0.0
Use of GLP1 analogue prior to or initiated at index date (n, %)	2565 (3.6)	956 (3.5)	1609 (3.7)	0.0
Lipid-lowering drug (n, %)	45,427 (64.4)	16,719 (61.6)	28,708 (66.1)	0.0
Antihypertensive drug (n, %)	60,376 (85.6)	23,636 (87.1)	36,740 (84.6)	0.0
Antiplatelet or anticoagulant drug (n, %)	45,923 (65.1)	16,932 (62.4)	28,991 (66.8)	0.0
Latest diagnosis at index date (n, %)				0.0
CVD	32,178 (45.6)	13,147 (48.5)	19,031 (43.8)	
CVD and T2D	579 (0.8)	176 (0.6)	403 (0.9)	
T2D	37,781 (53.6)	13,810 (50.9)	23,971 (55.2)	
CVD type (n, %)				0.0
Ischemic heart disease	34,443 (48.8)	12,081 (44.5)	22,362 (51.5)	
Heart failure	10,982 (15.6)	4494 (16.6)	6488 (14.9)	
Peripheral arterial disease	8583 (12.2)	3410 (12.6)	5173 (11.9)	
Cerebrovascular disease	16,530 (23.4)	7148 (26.3)	9382 (21.6)	

*SGLT2* Sodium-Glucose Transport Protein 2; *GLP1* Glucagon-Like Peptide 1; *DPP4* Dipeptidyl Peptidase-4; *HbA1c* Hemoglobin A1c (International Federation of Clinical Chemistry; National Glycohemoglobin Standardization); *LDL* low-density lipoprotein cholesterol; *GFR* glomerular filtration rate

^a^There were missing values in education level and the biochemical variables i.e. HbA1c, LDL, and eGFR. We imputed 10 datasets for these variables

### Time-to-initiation of cardioprotective glucose-lowering drugs

**Fig. 1:**
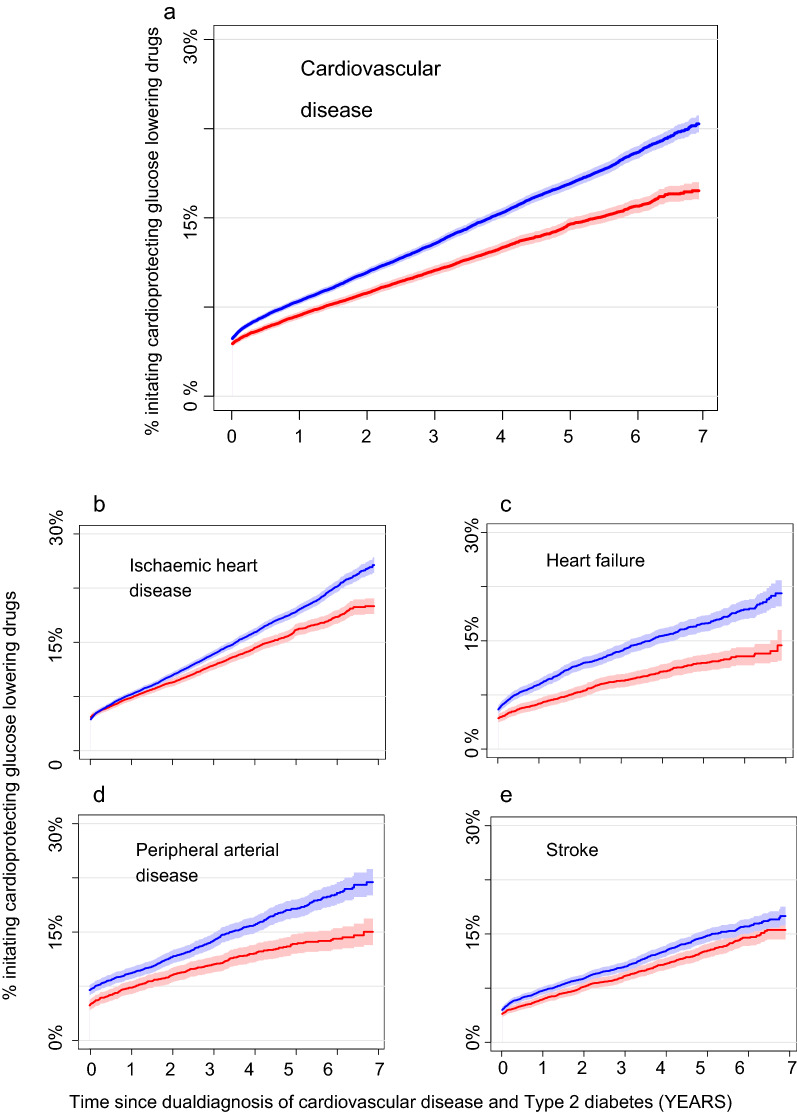
Time to initiation of cardioprotective glucose-lowering drugs in male and female patients with a first dual diagnosis of type 2 diabetes and cardiovascular disease. **A** Male and female patients with a new-onset dual diagnosis of type 2 diabetes and any cardiovascular disease. **B** Male and female patients with new-onset T2DM and ischemic heart disease. **C** Male and female patients with new-onset T2DM and stroke. **D** Male and female patients with new-onset T2DM and peripheral artery disease. **E** Male and female patients with new-onset T2DM and heart failure. Prevalent users of cardioprotective GLDs are included in graph at time = 0. Red = women, blue = men. *GLP*-*1RA* glucagon-like peptide-1 receptor agonist; *SGLT2* sodium-glucose co-transporter-2

Figure 1A shows cumulative incidence curves (CIP) of cardioprotective GLD initiation for female and male patients with a new dual diagnosis of T2DM and CVD. The CIP-curves did not start in 0%, as we included those who were prevalent users by inclusion. In spite of similar prevalence by inclusion i.e. a comparable starting point at day 0 (prevalent users), curve slopes continued to diverge during follow-up with a lower incidence of GLD initiation among female patients. Seven years after a dual diagnosis of T2DM and CVD, 17.3% (95%CI: 16.6–18.0%) of women patients had initiated a cardioprotective GLD, whereas the corresponding CUP among men was 23.0% (95%CI: 22.3–23.8%). To reach a CUP of 10%, women were approximately one year slower than men i.e., men reach a CUP of 10% within two years, while it took approximately three years for women.

Figure 1b-e shows cumulative incidence curves for women and men stratified by CVD type. In general, initiation of cardioprotective GLDs occurred at a lower rate in women compared with men with different slopes and intersections depending on CVD type. In ischemic heart disease, female and male incidence curves diverged with lower incidence of GLD initiation among women, however curves were confluent from baseline until year 2 after index date. In heart failure, incidence curves between female and male patients showed a similar pattern to the entire cohort (any CVD, Fig. 1a) with diverging curves during follow-up. Among patients with PAD, there was a small difference in prevalent GLD users at day = 0 and the slope of initiation curves was visually similar the first 2 years. Hereafter, the curve slope of men seemed to increase more than observed in women. Among stroke patients, initiation curves were similar in men and women.

For both genders, the CUPs at the end of follow-up were highest for ischemic heart disease and lowest for stroke.

### 1-year cumulative user proportion of cardioprotective glucose-lowering drugs

1-year CUPs of cardioprotective GLDs were lower in female patients with heart failure, PAD and stroke compared with male patients (Additional file 2: Fig. S2a, 1-year CUP female vs. male: heart failure 6.3% [95% CI 5.6–7.0%] vs 8.9% [95% CI 8.2–9.6%); PAD 7.2% [95% CI 6.4–8.2%] vs 9.2% [95% CI 8.5–10.1%); stroke 5.8% [95% CI 5.4 – 6.5%] vs. 7.2% [95% CI 6.7–7.7%]), whereas no gender difference was observed among patients with ischemic heart disease. In patients with heart failure, the gender difference was driven by higher 1-year CUPs of both SGLT2-inhibitors and GLP1 analogues, whereas the gender difference in PAD patients was mainly driven by the use of GLP-1 analogues (Additional file 2: Fig. S2b, c). Conversely, gender differences in stroke patients were mainly driven by initiation of SGLT2 inhibitors (Additional file 2: Fig. S2b, c).

### Initiation rates and initiation rate ratios for female and male patients

Among patients naïve of cardioprotective GLDs at index date, a 24% lower crude initiation rate was observed in female patients compared with male patients (Table 2). Upon adjustment, this association diminished to 14% (model 1) and 8% (model 2) lower initiation rate in women (Table 2).

**Table 2:** Initiation rates and initiation rate ratios for female and male patients

	Persons	Individuals initiating GLD	Person Years (PY)	Initiation rate (per 1000 PY)	Crude initiation rate ratio (95% CI)	Adjusted 1 initiation rate ratio (95% CI)	Adjusted 2 initiation rate ratio (95% CI)
Cardiovascular disease							
Male	41,333	4261	113,195	37.64 (36.53–38.79)	–	–	–
Female	25,949	2015	70,061	28.76 (27.53–30.04)	0.76 (0.72–0.81)	0.86 (0.82–0.91)	0.92 (0.87–0.97)
Ischemic heart disease							
Male	21,416	2558	62,405	40.99 (9.43–42.61)	–	-	-
Female	11,530	1092	34,429	31.72 (29.89–33.66)	0.77 (0.72–0.83)	0.85 (0.79–0.91)	0.91 (0.85–0.98)
Heart failure							
Male	6137	592	14,441	41.00 (37.82–44.43)	–	–	
Female	4305	258	9772	26.40 (23.37–29.83)	0.64 (0.56–0.75)	0.81 (0.70–0.94)	0.85 (0.73–1.00)^a^
Peripheral arterial disease							
Male	4813	421	12,827	32.82 (29.83–36.11)	–	–	
Female	3247	218	8663	25.16 (22.04–28.73)	0.77 (0.65–0.90)	0.88 (0.75–1.04)	0.92 (0.78–1.09)
Stroke							
Male	8967	690	23,522	29.33 (27.22–31.61)	–	–	–
Female	6867	447	17,196	25.99 (23.69–28.52)	0.89 (0.79–1.00)	0.97 (0.86–1.10)	1.06 (0.93–1.20)

Adjustment 1: Age at dual diagnosis, diabetes duration, calendar year and educational level

Adjustment 2: adjustment 1 + HbA1c, LDL, eGFR, lipid lowering medication, and antihypertensive medication

*HbA1c* Hemoglobin A1c; *LDL* low-density lipoprotein cholesterol; *GFR* glomerular filtration rate

^a^Upper limit of 95% confidence interval < 1.00

Gender modified the association between CVD type and cardioprotective GLD initiation (p = 0.01). Regardless of CVD type, crude initiation rate ratios were lower in female patients than male patients (Table 2). The magnitude of associations was attenuated by adjustment in all CVD types, but a statistically significant difference between women and men remained in patients with ischemic heart disease and heart failure (Table 2).

**Fig. 2:**
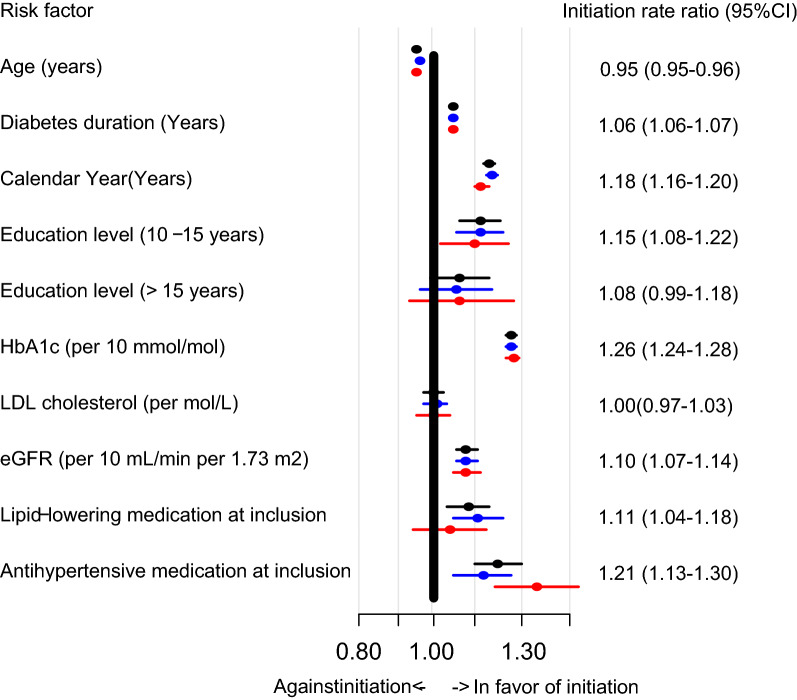
Forest plot of gender and risk factors important for cardioprotective GLD initiation. Black: Entire cohort (female and male patients), red = women, blue = men. Initiation rate ratio numbers are listed for the entire cohort. *HbA1c* Hemoglobin A1c; *LDL* low-density lipoprotein cholesterol; *GFR* glomerular filtration rate

In the entire study population, age was a negative predictor of cardioprotective GLD initiation, whereas positive predictors included calendar year of inclusion, higher education level, diabetes duration, higher HbA1c levels, higher e-GFR and use of lipid-lowering and antihypertensive drugs (Fig. 2). In gender-stratified analysis, we found a similar direction for these predictors in both women and men, however, antihypertensive medication was a stronger predictor in women, whereas lipid-lowering medication was stronger in males. In a sensitivity analysis using non-imputed data, we found similar results (data not shown).

For both genders, the initiation rate declined with age i.e. the initiation rate per 1000 PY in males < 60 years was 73.0 per 1000 PY (95% CI 69.8–76.3), age 60–70 years it was 39.1 (95% CI 37.2–41.2), age 70–80 it was 20.9 (95% CI 19.4–22.5) and in males > 80 years, it was 7.9 per 1000 PY (95%CI 6.6–9.5). Correspondingly, in females < 60 years, the initiation rate per 1000 PY was 67.9 (95% CI 63.5–72.5), in females between 60 and 70 years it was 34.5 (95% CI 31.7–37.3), age 70–80 it was 18.6 (95% CI 16.9–20.5) and in females above 80 years it was 5.0 (95% CI 4.0–6.2). While we found no difference in the adjusted initiation rate ratio between men and women < 60 years (IRR 1.0 [95% CI 0.9–1.1]), the initiation rate declined faster in women than in men i.e. the initiation rate ratios in the remaining three age strata were: age 60–70 years, IRR 0.9 [95% CI 0.8–1.0]; age 70–80 years, IRR 0.9 [95% CI 0.8–1.0]; age > 80 years, IRR 0.7 [95% CI 0.5–0.9]), respectively.

## Discussion

This nationwide cohort study shows that despite national and international guidelines, the initiation of cardioprotective GLDs remained relatively low among all patients with a new dual diagnosis of T2DM and CVD in Denmark. Female patients had lower cumulated user proportions compared to male patients. In crude and adjusted regression analyses, we found that treatment initiation rates with GLDs were lower for women compared with men. The lower initiation rate of GLDs was mainly apparent in older individuals and in women with ischemic heart disease and heart failure.

### Gender differences in CVD drug initiation

Optimal medical treatment (e.g., the proportion of patients receiving antiplatelets, antihypertensive drugs, statins, and/or cardioprotective GLDs) in patients with CVD is only achieved in a small subset of patients [28]. Most often, undertreatment is explained by suboptimal medicine adherence, and multiple clinical and observational studies have shown lower adherence to prescribed CVD medication in women than in men (as summarized in a recent editorial by Sederholm et al. [4]). Specifically in patients with type 2 diabetes, it has recently been shown, that female patients have poorer guideline-recommended risk factor control compared with male patients [29]. Subclinical atherosclerosis is more prevalent in non-diabetic women than men with signs of insulin resistance and uncontrolled risk factors [30], and may indicate why female T2DM patients face a higher risk of CVD than men [2] Lower rates of drug initiation may be an important part of under treatment that is often overlooked.

US and German studies have explored baseline characteristics of cardioprotective GLD initiators and found that the majority of SGLT2 inhibitor initiators are male (57–66%), whereas the female and male proportion among GLP1 initiators is almost equally balanced [31, 32]. Importantly, these studies did not report data on cardioprotective GLD naïve patients eligible for treatment. An American study by Arnold et al. identified patients who fulfilled in/exclusion criteria for the EMPAREG (cardiovascular outcome trial of empagliflozin) and LEADER study (cardiovascular outcome trial of liraglutide) and found that initiators of an SGLT2 inhibitor were more likely to be male (66.6%) compared to non-initiators (63.4), whereas no difference was found between initiators and non-initiators of GLP1 analogous [33]. Importantly, comparisons between women and men were not adjusted for confounders and, furthermore, due to selection bias inherent in clinical trials, these study results may not be generalizability to a broad population of patients with T2DM and CVD [34]. We present a longitudinal cohort study on all patients with a new-onset dual diagnosis of T2DM and CVD (2012–2018) among the entire Danish population and thus, our results apply to a broad spectrum of patients eligible for treatment with a cardioprotective GLD.

### Potential barriers for CVD initiation in women and men

Gender differences in CVD treatment may be explained by a number of factors considered important for medication adherence; e.g. the WHO lists patient-related-, socioeconomic-, health system-, therapy-, and condition-related factors [35]. Interestingly, in a recent meta-analysis of eleven randomized controlled trials, drug discontinuation was higher among women than men, even after adjustment of multiple confounders, including age, comorbidities, polypharmacy, ethnicity and perceived side effects [3]. Other studies have identified socio-economic status and health literacy as important barriers to CVD drug adherence [36–38]. In our study, we investigated a number of barriers to GLD initiation that may confound the comparison between women and men. After adjustment for “non-modifiable” factors (model 1): age, diabetes duration, calendar year and education level, a substantially lower rate of cardioprotective GLD initiation among women was observed. In a previous study, we found that prescription patterns have changed during the study period [21], and therefore we added calendar year to the model. With further adjustment for modifiable factors (HbA1c, LDL cholesterol, eGFR, antihypertensive medication and lipid-lowering drug treatment), the point estimate was reduced from 24% to 8% in favor of men. Our results thus indicate that GLD initiation is partly explained by these factors and that these factors/barriers are largely of similar importance in men and women. As expected, higher Hba1C was associated with higher initiation rates as the initiation of any GLD during the majority of the study period was partly driven by glycemic targets. Similarly, we expected that higher eGFR was associated with higher initiation rates, as the nephroprotective effects of SGLT2-inhibitors observed in CREDENCE was first published in 2019 [39]. The use of other CVD medications was positively associated with GLD initiation, which may have several explanations: Likely, the use of other CVD medications reflects the severity of CVD and thus the motivation from clinician and patient to prescribe and use aggressive risk factor control. Another plausible explanation may be that the use of other CVD medications is linked to the willingness of a patient to initiate and adhere to multiple CVD medications. Finally, in age-stratified analysis, we found that GLD initiation rates declined with age and to a larger extent in women. This may also relate to the same mechanisms as above; that men have more severe CVD with age or is perceived to have higher risk of recurrent CVD with age, thus cardioprotective drugs are more often prescribed to male patients.

As this is an observational study, we can only speculate on what drives the remaining difference in GLD´s initiation in men and women. Fear of medication side effects (from either clinician or patient) may affect women and men differentially. For example, SGLT2 inhibitors increase the risk of genital infections in both genders [40], but this information may affect the decision of SGLT2 inhibitor initiation differently with a lower possibility of initiation among women. Complex cultural factors that affect both clinicians (interaction of patient gender on clinician-patient relationship) and patients (perception of disease) may affect the chance of GLD initiation in favor of men. Interestingly, Lau et al. found that gender disparities in CVD drug discontinuation depended on geographical region (large gender differences in North America and Europe vs. no gender difference in South/Central America) ^3^, and this could imply that some of the same factors are relevant in our study.

### Initiation of cardioprotective GLDs in specific CVD subtypes

Regardless of CVD type, initiation rates were lower among women than men, however after adjustment for potential confounders the associations were strongest and only remained significant for ischemic heart disease and heart failure. It is important to recognize that patients with ischemic heart disease and heart failure showed overall higher CUPs and initiation rates compared with PAD and stroke. Thus, potential gender differences in GLD initiation among patients with PAD or stroke may be harder to detect due to a lower number of initiators in these groups. Different barriers to cardioprotective GLD initiation specific to CVD type may however exist. For example, CVD type may be linked to other comorbidities, CVD sequelae or reduced life expectancy that differ between genders and that may differentially affect the patient or clinician motivation for GLD initiation. For example, male heart failure patients have higher mortality rates compared with women, which may reflect a more severe disease in men, thus increasing the likelihood of GLD initiation [41]. On the other hand, female PAD patients showed increased high-risk anatomical features and increased risk of postoperative bleeding than men in aortoiliac occlusive disease, which be a factor of importance when interpretating the similar GLD initiation rates in women and men with PAD [42]. Continuity of patient care (e.g., the transition between secondary and primary sectors) also differ according to CVD type and it may contain inherent factors favorizing males (for example, rehabilitation programs may be designed to optimize compliance with a focus on male patient needs).

### Strengths and limitations

The broad inclusion of all patients with T2DM and CVD does not take contra-indications for cardioprotective GLDs into account, and thus, a small part of the study population may not have been eligible for cardioprotective GLD treatment according to current drug labels. This may have biased our estimates if these factors were more prevalent among women than men, e.g. if women had a higher prevalence of hypotension and thus did not qualify for SGLT2 inhibitors. Moreover, the accuracy of registry data is important for the selection of study participants and outcome registration, however previous studies have found high positive predictive values for the CVD diagnosis and procedural codes used in this study [24, 25], while no off-label prescriptions of GLDs is expected as a doctor prescription is necessary for these drugs in Denmark. Furthermore, it is unlikely that potential information bias introduced by inaccurate registry data would affect the comparison between women and men to a considerable extent, as we would expect any bias in registry data to be non-differential. In our data set, we had missing values in education level and the biochemical variables and in order to limit selection bias and loss of statistical power in adjusted analysis, we used imputed data. We found similar results in sensitivity analysis using non-imputed data (data not shown). Moreover, we did not have access to data on BMI and lifestyle factors, which may be important confounding factors for the observed gender differences (e.g. in Denmark, smoking is more prevalent among men than women [43], and it may increase the chance for cardioprotective GLD initiation). Another important point is that the outcome data from the National Database of Reimbursed Prescriptions reflects dispenses of GLDs, thus we cannot differentiate whether low initiation rates were due to clinical inertia (no doctor prescriptions) or patient barriers to redeem a prescription. Finally, due to a major update of the Danish National Patient Register, data was only available until Dec 2018. The use of SGLT2 inhibitors and GLP analogues however, is still in transition as clinical studies point to broadened indications (e.g. nephropathy and overweight management) and future studies are warranted to explore how this may affect gender differences in GLD initiation.

## Conclusion

Women with a new dual diagnosis of T2DM and CVD have lower initiation rates and lower CUPs of cardioprotective GLDs compared with men. This was mainly driven by gender differences in older patients and in patients with ischemic heart disease and heart failure. Women with new onset T2DM and CVD are approximately 1 year slower than men to reach a 10%-CUP of cardioprotective GLDs.

## Supplementary Information

**Additional file 1: Figure S1.** Title: Study design. *Laboratory values were assessed up until 30 days after the index date.

**Additional file 2: Figure S2a–c.** Proportions in treatment with SGLT2-inhibitors and GLP1 analogues within. 1 year after diagnosis of type 2 diabetes and cardiovascular disease in female and male patients. A, any cardioprotective GLD. B, SGLT2-inhibtors. C, GLP1-analouges. Red = women, blue = men. GLP-1RA, glucagon-like peptide 1 receptor agonist; SGLT2, sodium glucose co-transporter-2.

**Additional file 3: Table S1.** ATC codes for drugs of interest including glucose-lowering drugs. **Table S2.** Codes for cardiovascular disease.

## Data Availability

The dataset was derived from national health registries and cannot be shared publicly. The data can be accessed through authorization from Danish regulatory authorities.

## Abbreviations

ATC: Anatomical therapeutic chemical
CIP: Cumulative incidence proportion
CUP: Cumulative user proportion
CVD: Cardiovascular disease
EASD: European Association for the Study of Diabetes
eGFR: Estimated glomerular filtration rate
GLD: Glucose-lowering drug
GLP1: Glucagon-like peptide 1
HbA1c: Hemoglobin A1c
ICD: International classification of diseases
LDL: Low-density lipoprotein
SGLT-2: Sodium glucose co-transporter 2
T2DM: Type 2 diabetes
