# Supplementary material for: Gender disparities in time-to-initiation of cardioprotective glucose-lowering drugs in patients with type 2 diabetes and cardiovascular disease: a Danish nationwide cohort study
Source: Cardiovasc Diabetol. 2022 Dec 10;21:279. doi: 10.1186/s12933-022-01713-3 (PMC9737743; doi:10.1186/s12933-022-01713-3)
Supplement: Supplementary file 3 — Additional file 3: Table S1. ATC codes for drugs of interest including glucose-lowering drugs. Table S2. Codes for cardiovascular disease. [file 12933_2022_1713_MOESM3_ESM.docx]

**Table S1** ATC codes for drugs of interest including glucose-lowering drugs

| **Drug class** | **ATC codes in database** |
| --- | --- |
| SGLT2-inhibitors | **A10BK,**  **A10BD15 - A10BD24,**  **A10BX09, A10BX11, A10BX12** |
| GLP1 receptor agonists | **A10BJ, A10AE5,**  **A10BX04, A10BX07,**  **A10BX10, A10BX13, A10BX14** |
| DPP4 inhibitors | **A10BD, A10BH** |
| Biguanides | **A10BA** |
| Sulfonylureas | **A10BB** |
| Glitazones | **A10BG** |
| Insulin and analogues | **A10A** |
| Metiglinides | **A10BX02, A10BX03, A10BX08** |
| Combinations of oral glucose lowering drugs | **A10BD** |
| Antihypertensive drugs | **C02CA, C03, C07, C08, C09, G04CA** |
| Lipid-lowering drugs | **C10** |
| Antithrombotic drugs | **B01AA, B01AC, B01AE07, B01AF, N02BA01** |

**Table S2** Codes for cardiovascular disease

| **Ischaemic Heart Disease** | **ICD-10 code** | **Title** |
| --- | --- | --- |
|  | I20 | Angina pectoris |
|  | I21 | Acute myocardial infarction |
|  | I22 | Subsequent myocardial infarction |
|  | I23 | Certain current complications following acute myocardial infarction |
|  | I24 | Other acute ischaemic heart diseases |
|  | I25 | Chronic ischaemic heart disease |
|  | **NCSP Procedural codes** |  |
|  | KFNA | Anastomosis between arteria mammaria interna and coronary artery |
|  | KFNB | Anastomosis between arteria gastroepiploica interna and coronary artery |
|  | KFNC | Aorto-coronary bypass operation |
|  | KFND | Aorto-coronary bypass operation with prothesis |
|  | KFNE | Coronary bypass operation with artery transplant |
|  | KFNF | Coronary thrombectomy |
|  | KFNG | Dilation and revascularization of coronary artery |
|  | KFNH20 | Reconstruction of coronary artery with bypass |
|  | KPAU74 |  |
|  | | |
| **Cerebrovascular Disease** | **ICD-10 code** | **Title** |
|  | I60 | Subarachnoid haemorrhage |
|  | I61 | Intracerebral haemorrhage |
|  | I63 | Cerebral infarction |
|  | I64 | Stroke, not specified as haemorrhage or infarction |
|  | **NCSP Procedural codes** |  |
|  | KAAL10 | Intracranial endovascular thrombolysis |
|  | KAAL11 | Intracranial intraarterial thrombectomy |
|  | | |
| **Peripheral artery disease** | **ICD-10 code** | **Title** |
|  | DI 74 | Arterial embolism and thrombosis |
|  | DI 702 | Atherosclerosis of arteries of extremities |
|  | DI739A | Claudicatio intermittens |
|  | **NSCP procedural codes** |  |
|  | KPAE | Thrombectomy and embolectomy of the truncal aorta and related branches |
|  | KPAF | Endarterectomy of the truncal aorta and related branches |
|  | KPAU74 | Thrombectomy and embolectomy of arteria carotis, arteria subclavia or arteria axillaris |
|  | KPBE | Thrombectomy and embolectomy of the arteries of the upper extremity |
|  | KPBF | Endarterectomy of the arteries of the upper extremity |
|  | KPCE | Thrombectomy and embolectomy of the visceral arteries |
|  | KPCF | Endarterectomy of the visceral arteries |
|  | KPCU74 | Thrombectomy and embolectomy of bypass of the visceral arteries |
|  | KPDE | Thrombectomy and embolectomy of the infrarenal aorta and illical arteries |
|  | KPDF | Endarterectomy of the infrarenal aorta and illical arteries |
|  | KPDH | Bypass surgery on infrarenal aorta and ilical arteries |
|  | KPEE | Thrombectomy and embolectomy of arteria femoralis and its branches |
|  | KPEF | Endarterectomy of arteria femoralis and its branches |
|  | KPEH | Bypass surgery on arteria femoralis and its branches |
|  | KPEU74 | Thrombectomy or embolectomy on bypass from arteria femoralis arteria poplitea |
|  | KPFE | Thrombectomy and embolectomy of arteria poplitea and lower leg arteries |
|  | KPFF | Endarterectomy of arteria poplitea and lower leg arteries |
|  | KPFH | Bypass surgery on arteria poplitea and lower leg arteries |
|  | KPFU74 | Thrombectomy or embolectomy on bypass from arteria femoralis or arteria poplitea to the lower extremity arteries |
|  | KPGH | Extra-anatomical bypass |
|  | KPGU74 | Thrombectomy or embolectomy of extra-anatomical bypass |
|  | | |
| **Heart Failure** | **ICD-10 code** | **Title** |
|  | I50 | Heart failure |
|  | I110 | Hypertensive heart disease with (congestive) heart failure |
|  | I130 | Hypertensive heart and renal disease with (congestive) heart failure |
|  | I132 | Hypertensive heart and renal disease with both (congestive) heart failure and renal failure |

Legend: ICD = International Classification of Diseases, NSCP = Nordic Classification of Surgical Procedures
